# Childhood Trauma and Functional Connectivity between Amygdala and Medial Prefrontal Cortex: A Dynamic Functional Connectivity and Large-Scale Network Perspective

**DOI:** 10.3389/fnsys.2017.00029

**Published:** 2017-05-11

**Authors:** Josh M. Cisler

**Affiliations:** Department of Psychiatry, University of Wisconsin–Madison, MadisonWI, USA

**Keywords:** early life trauma, amygdala, functional connectivity, dynamic functional connectivity

## Abstract

Altered functional connectivity (FC) between the medial prefrontal cortex (mPFC) and amygdala is widely implicated as a neural mechanism explaining risk for psychopathology among those exposed to early life trauma. Nonetheless, contemporary neuroimaging research has shifted toward large-scale network models of brain function, and it is not clear how this common bi-nodal finding fits into larger-scale network models. Here, using dynamic functional connectivity (DFC) approaches combined with large-scale network analyses, the larger role of bi-nodal FC between mPFC and amygdala among a sample of adolescent girls is investigated. The sample was comprised of 30 healthy control girls and 26 girls exposed to either physical or sexual assault who underwent a resting-state scan during 3T MRI. DFC using a sliding window approach was used to create weighted, undirected, graphs from the resting-state data following parcellation with a 215 regions-of-interest (ROI) atlas. Using *a priori* ROI, the predicted finding of lessor FC between mPFC and amygdala as a function of early life trauma was replicated in this sample. By contrast, early life trauma was associated with greater large-scale network modularity. Using a dynamic FC approach, it is also demonstrated that within-subject variability in this bi-nodal FC closely tracks within-subject fluctuations in large-scale network patterns, including connectivity between a limbic and default mode network (in which the amygdala and mPFC nodes belong, respectively) as well as overall modular organization. These results suggest that bi-nodal FC, such as amygdala-mPFC FC, may generally reflect larger-scale network patterns. Future research is necessary to understand whether these associations between nodal FC and large-scale network organization better reflect top-down processes (larger-scale network organization drives bi-nodal FC) or bottom-up processes (bi-nodal FC drives larger-scale network organization) and the related impact of early life trauma.

## Introduction

A central role for the functional connectivity (FC) between the amygdala and medial prefrontal cortex (mPFC) is ubiquitous in neurocircuitry models of trauma and PTSD (Rauch et al., 2006; Pitman et al., 2012; Admon et al., 2013). Within the context of these models, amygdala activity is conceptualized as reflecting threat processing, and mPFC activity is conceptualized as reflecting an emotion regulation/inhibition process (Rauch et al., 2006; Pitman et al., 2012; Admon et al., 2013). Weakened FC between these nodes is conceptualized as weakened top-down control of the amygdala by the mPFC and is theorized to mediate over-expression of negative affectivity and under-expression of emotion regulation (e.g., implicit emotion regulation, fear extinction learning, etc) among individuals with PTSD (Rauch et al., 2006; Patel et al., 2012; Pitman et al., 2012). Consistent with these models’ predictions, there is consistent data suggesting that altered amygdala-mPFC FC at rest scales with degree of early life trauma and additionally predicts internalizing symptoms and cortisol levels (Burghy et al., 2012; Herringa et al., 2013; Pagliaccio et al., 2015). As such, the strength of amygdala-mPFC FC appears to be a potent mechanism explaining risk for PTSD and related affective psychopathology among those exposed to early life trauma.

The purpose of the current study is to examine this common finding of altered amygdala-mPFC connectivity among those exposed to early life trauma within the context of large-scale network models of human brain function. Network-level approaches to human functional brain organization demonstrate spatially distributed networks consisting of brain regions with greater within-network temporal covariance compared to between-network temporal covariance (Bullmore and Sporns, 2009; Bressler and Menon, 2010; Rubinov and Sporns, 2010; Menon, 2011). The spatial organization of these networks is remarkably consistent across studies, which demonstrate canonical motor, salience, default mode, frontoparietal, and visual networks (Damoiseaux et al., 2006; Smith et al., 2009). From the perspective of larger-scale networks in the human brain, it is interesting to consider how to conceptualize a single bi-nodal connection, such as between the amygdala and mPFC. That is, the mPFC often is found to be part of a default mode network (DMN) (Raichle, 2015) while the amygdala is found to be part of a limbic network (Smith et al., 2009; Onoda and Yamaguchi, 2013; Cisler et al., 2016; Schlesinger et al., 2017). It is relevant to mention here that, while the amygdala is often regarded intuitively as belonging to the salience network (Bressler and Menon, 2010), the salience network is most consistently characterized by dorsal anterior cingulate cortex and bilateral anterior insular cortex (Uddin, 2015). Nonetheless, the interesting aspect of focusing on bi-nodal connectivity remains: if the two regions belong to different networks, does altered connectivity between them simply reflect connectivity between the larger networks in which the nodes belong? Similarly, modularity is a growing concept in network-level approaches to brain organization in the past few years and refers to the degree to which a network can be sub-divided into functionally specialized modules (Hartwell et al., 1999; Meunier et al., 2009; Rubinov and Sporns, 2010; Onoda and Yamaguchi, 2013; Cisler et al., 2016; Schlesinger et al., 2017). Modularity is formally defined as the difference between the number of observed connections within modules and the number of connections expected given chance distribution (Newman, 2006). Greater values reflect networks with more cleanly segregated modules. From this perspective, the intriguing question regarding amygdala-mPFC FC is whether degree of connection between these nodes belonging to different modules only reflects larger modular organization patterns of the network. For example, a network with greater separation between modules (i.e., more pronounced modular organization) would by definition be expected to have weaker connections between nodes belonging to different modules. As such, perhaps the weaker amygdala-mPFC observed among those exposed to early life trauma simply reflects greater overall modularity.

One means of addressing these questions regarding the role of bi-nodal connectivity within larger-scale network patterns is to examine dynamic functional connectivity (DFC) (Calhoun et al., 2014). DFC is a relatively newer approach to FC analysis and is based on the concept that the degree to which brain regions are connected is not static and instead connectivity between brain regions can fluctuate depending on various factors such as context, experimental task, alertness, etc (Hutchison et al., 2013; Calhoun et al., 2014; Gonzalez-Castillo et al., 2015). This approach frequently uses a sliding window in which FC is defined repeatedly across the scan (Allen et al., 2014). For example, using a window length of 30 TRs, one would define connectivity between regions (or voxels or networks) from TR 1 to TR 30, then shift the window forward and redefine connectivity between TR 6 and 35, and so forth throughout the scan. Using this approach to address the current questions allows for a within-subject analysis of the degree to which temporal fluctuations in amygdala-mPFC connectivity track temporal fluctuations in larger-network patterns. At a second-level (group-level) of analysis, the consistency of these within-subject effects can be tested, as can any moderating role of early life trauma-exposure on these analyses across network-levels. Accordingly, the DFC approach is used here to test the alternative hypothesis that amygdala-mPFC connectivity during rest reflects larger patterns of network organization.

## Materials and Methods

### Participants and Assessment

The initial sample consisted of 68 adolescent girls, aged 11–17. Twelve participants were removed for either head motion (*n* = 8; detailed below) or poor coverage (*n* = 4; e.g., excessive OFC dropout, lack of coverage in dorsal motor cortex, etc) during image acquisition, leaving a final sample of 56 adolescent girls. **Table 1** describes clinical and demographic data for the sample. The control group consisted of 30 girls with no history of trauma-exposure, no current mental health disorders, and were not currently taking psychotropic medication. The assaulted adolescent group consisted of 26 girls who experienced either physical or sexual assault.

**Table 1:** Clinical and demographic characteristics of the sample.

Variable	Control (*n* = 30)	Assaulted (*n* = 26)	Group difference *p*-value
Age	14.7 (1.92)	15.2 (1.52)	0.3
Ethnicity	73% Caucasian 23% African American 3% other	58% Caucasian 35% African American 4% Asian 4% Native American	0.23
Direct assaults	–	3.39	–
PTSD diagnosis	–	38%	–
UCLA PTSD RI	2.0 (4.8)	21 (17.1)	<0.001
Childhood Trauma Questionnaire total score	37.7 (9.4)	57.6 (15.5)	<0.001
Verbal IQ	107.53 (19.07)	99.35 (13.67)	0.07
Psychotropic medication	–	42%	–
Short mood and feelings questionnaire	3.0 (3.2)	8 (7.0)	0.001

*UCLA PTSD RI = UCLA PTSD Reaction Index.*

All participants’ mental health was assessed with either the K-SADS (Kaufman et al., 1997) (*n* = 36) or MINI-KID (Sheehan et al., 2010) (*n* = 22). Both are widely used structured clinical interviews for most Axis I disorders found in childhood and adolescence. Assaultive trauma histories were characterized using the trauma assessment section of the National Survey of Adolescents (NSA) (Kilpatrick et al., 2000, 2003), a structured interview used in prior epidemiological studies of assault and mental health functioning among adolescents that uses behaviorally specific dichotomous questions to assess sexual assault, physical assault, severe abuse from a caregiver, and witnessed violence. Participants also completed a more inclusive assessment of childhood maltreatment via the Childhood Trauma Questionnaire (CTQ) (Bernstein and Fink, 1998), a widely used self-report measure assessing separate physical abuse, physical neglect, emotional abuse, emotional neglect, and sexual abuse domains of childhood trauma. Analyses here used multiple regression in which each of these CTQ subscales were entered simultaneously as predictors, allowing for an investigation of unique variance in brain function attributable to the individual subscale. Higher scores on the CTQ represent more severe trauma histories. The assessments also included measures of verbal IQ (receptive one word picture vocabulary test; Brownell, 2000), PTSD symptom severity (UCLA PTSD Reaction Index; Steinberg et al., 2004, 2013), and depression severity (Short Mood and Feelings Questionnaire; SMFQ; Angold et al., 1995).

### Resting-State Task

Participants were presented with a fixation cross and instructed to try and keep their eyes open and look at the cross and to let their minds wander naturally and not try to think about anything specific. The resting-state scan lasted 450 s.

### MRI Acquisition

For 36 participants (*n* = 17 directly assaulted adolescents), a Philips 3T Achieva X-series MRI system with an 8-channel head coil (Philips Healthcare, USA) was used to acquire imaging data. Anatomic images were acquired with a MPRAGE sequence (matrix = 256 × 256, 160 sagittal slices, TR/TE/FA= 2600ms/3.02ms/80, final resolution = 1 mm × 1 mm × 1 mm resolution). Echo planar imaging (EPI) sequences were used to collect the functional images using the following sequence parameters: TR/TE/FA = 2000ms/30ms/900, FOV = 240 mm × 240mm, matrix = 80 × 80, 37 oblique slices (parallel to AC-PC plane to minimize OFC signal artifact), slice thickness = 3mm, interleaved acquisition sequence.

For 22 participants (*n* = 9 directly assaulted adolescents), image acquisition parameters were slightly different. A 32-channel head coil was used to acquire the imaging data. Anatomic images were collected using identical sequences and parameters. The EPI images were collected using identical parameters except slice thickness was 2.5 mm with a 0.5 mm gap in between slices and collected in an ascending order and resampled during preprocessing to a final resolution = 3 mm × 3 mm × 3 mm. Importantly for the present analyses, image acquisition methodology was not correlated with CTQ (*r* = 0.14, *p* = 0.3) nor did it differ between assaulted and control adolescents (χ^2^ = 0.03, *p* = 0.87).

### Image Preprocessing

Image preprocessing followed standard steps and was completed using AFNI software. In the following order, images underwent despiking, slice timing correction, deobliquing, motion correction using rigid body alignment, alignment to participant’s normalized anatomical images, spatial smoothing using a 8 mm FWHM Gaussian filter (AFNIs 3dBlurToFWHM that estimates the amount of smoothing to add to each dataset to result in the desired level of final smoothing), detrending, bandpass filtering with frequencies of 0.1 and 0.01 Hz, and rescaling into percent signal change. Images were normalized using the MNI 452 template brain. Following recent recommendations (Power et al., 2014; Siegel et al., 2014), we corrected for head motion related signal artifacts by using motion regressors derived from Volterra expansion, consisting of [*R R*^2^ *R*_t-1_ *R*^2^_t-1_], where *R* refers to each of the six motion parameters, and separate regressors for mean signal in the CSF and WM. This step was implemented directly after motion correction and normalization of the EPI images in the image preprocessing stream. Additionally, we censored TRs from the first-level analyses based on threshold of framewise displacement (FD) > 0.5. FD refers to the sum of the absolute value of temporal differences across the six motion parameters; thus, a cut-off of 0.5 results in censoring TRs where the participant moved, in total across the six parameters, more than ∼0.5 mm plus the immediately following TR (to account for delayed effects of motion artifact). Additionally, we censored isolated TRs where the preceding and following TRs were censored, and we censored entire runs if more than 50% of TRs within that run were censored, leading to the removal of eight participants from all analyses.

### fMRI Data Analysis

#### Group-Level Modular Brain Organization

First, the group-level modular brain organization for the sample was defined. For computational tractability, the 250 regions-of-interest (ROI) functional atlas was used (Craddock et al., 2012). After accounting for individual differences in spatial coverage across participants, 215 ROIs were retained in the parcellation. For each individual, the mean time course of voxels within each ROI was calculated, excluding voxels within an ROI that may be outside the brain for a given individual, resulting in a 225 × 215 matrix for each individual. These matrices were concatenated across participants and then correlated and *r*-to-*z* transformed, resulting in a single 215 × 215 square weighted matrix. The diagonals and negative values were set to zero (Rubinov and Sporns, 2010). The Brain Connectivity Toolbox (Rubinov and Sporns, 2010) implemented in Matlab was used to detect the community (module) structure of the group-level weighted matrix (the ‘community_louvain.m’ function). Given that the gamma resolution parameter for the Louvain algorithm is a free parameter that needs to be selected, a data-driven approach was used to identify the optimal resolution parameter that resulted in greatest partition similarity across repeated Louvain algorithm iterations. A range of resolution parameters between 1 and 2 in 0.1 increments was tested, and for each resolution parameter, the Louvain algorithm was conducted 250 times to identify 250 network partitions and the average Rand *z*-scored index of similarity (Traud et al., 2011) across these partitions was calculated. The results for the average Rand *z*-scored index of similarity are depicted in **Supplementary Figure S1**. While a resolution parameter = 1 was associated with marginally higher similarity across partitions compared to the next highest parameter of 1.7, the resolution parameter of 1 only identified 3 modules (motor cortex, visual cortex, with everything combined into a remaining module) that does not map onto canonical human brain functional networks. By contrast, a resolution parameter = 1.7 identified 10 modules that mapped onto well-known large-scale networks in the human brain. We accordingly selected the resolution parameter of 1.7 as the parameter that optimizes both similarity across partitions as well as correspondence with known functional networks.

**FIGURE 1:**
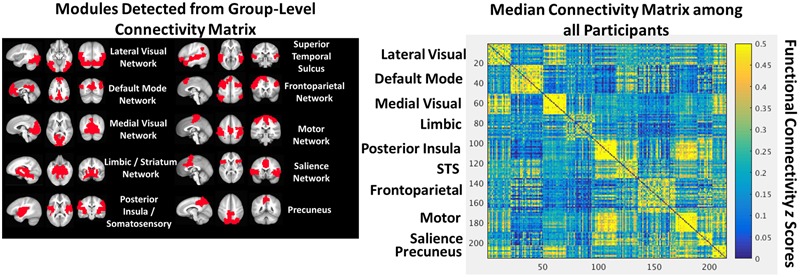
**The group-level community structure projected into anatomical space **(Left)**.** A heat map depicting the group-level connectivity matrix sorted by module **(Right)**.

We identified a stable partition with the 1.7 gamma resolution parameter using the procedure described by Cohen and D’Esposito, 2016. In this approach, the Louvain algorithm is performed 300 times, identifying 300 partitions. An agreement matrix is then calculated using the agreement_weighted.m function from the Brain Connectivity Toolbox, characterizing the degree of agreement across partitions. An iterative procedure then begins in which the Louvain algorithm is performed 300 times on this agreement matrix, a new agreement matrix is calculated, and this process repeats until either there is perfect agreement in partitions across iterations or performance (i.e., degree of agreement) stops improving across iterations. This approach resulted in a network partition with 10 modules that correspond well with known functional networks and is depicted in **Figure 1**.

#### Dynamic Functional Connectivity

In this work, a sliding window of 44 s (22 TRs) was used, shifting each window by 5 TRs, across the entire resting-state scan. Prior work has used a sliding window length of as small as 10 s (Thompson et al., 2013) and as long 180 s (Gonzalez-Castillo et al., 2015). The rationale here for a moderate-length sliding window length was to simultaneously maximize statistical power within the window (number of time points with which to correlate) while also maximizing statistical power for cross-level analyses (e.g., number of FC observations to correlate between the bi-nodal amygdala-mPFC FC and modularity *Q*-values). Importantly, amygdala-mPFC defined using a static approach was strongly related to median amygdala-mPFC FC across the sliding windows (*r* = 0.78, *p* < 0.001), as were static modularity *Q*-values and median modularity *Q*-values across the sliding windows (*r* = 0.93, *p* < 0.001), suggesting that the window length used here was not fundamentally altering the bi-nodal FC or the larger network organization patterns within or across subjects.

Within each individual, the 215 × 215 *r*-to-*z* transformed square weighted matrix was calculated at each window. This allowed modularity *Q*-values (again using the community_louvain.m function with a gamma value of 1.7) and between-module FC to be calculated at each window. Between-module FC was calculated within each individual using the group-level network partitions (**Figure 1**), with the module time course defined as the mean time course of all ROIs within the module.

Functional connectivity between the amygdala and mPFC was additionally calculated at each window using *a priori* ROIs identified in a meta-analysis of regions demonstrating altered functional activation in PTSD (Patel et al., 2012). This meta-analysis identified a single ROI in the left amygdala, which was used to place a 6 mm spherical ROI with MNI coordinates of *X* = -20, *Y* = 5, *Z* = -15. While the meta-analysis reported two rostral anterior cingulate clusters, only one was located in the pregenual anterior cingulate cortex (pgACC), and this cluster was used to define a 10 mm spherical ROI (10 mm to capture both left and right pgACC centered at MNI coordinates of *X* = 0, *Y* = 42, *Z* = 4). The mean time course of voxels within these ROIs were calculated within each individual and then FC between these ROIs was calculated at each window.

**FIGURE 2:**
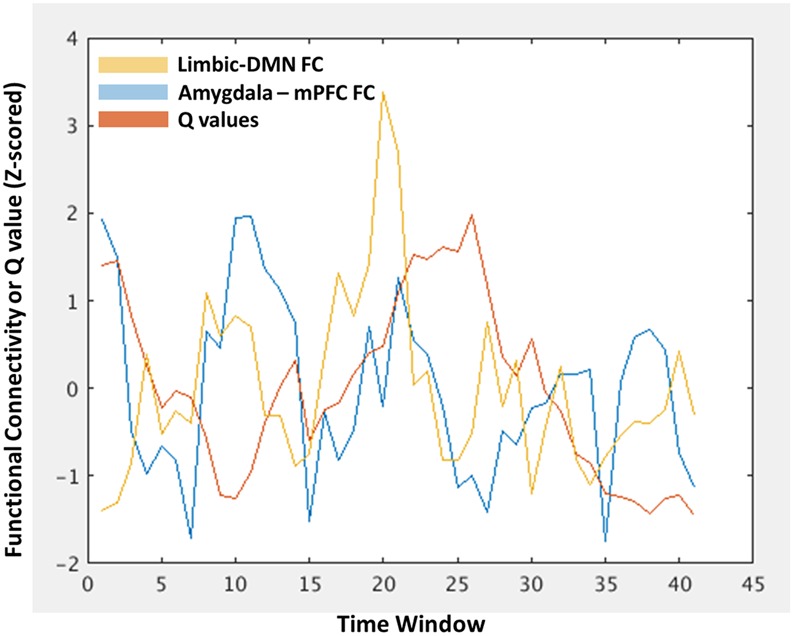
**An illustration of the within-subject characterization of modularity *Q*-values (red), limbic-DMN FC (orange), and amygdala-mPFC FC (blue) at each time window, allowing tests of window-by-window associations between these different levels of network analysis.** The *x*-axis represents each time window in which the functional connectivity or *Q*-value was estimated. The *y*-axis represents the functional connectivity index of *z*-scored *Q*-value.

This DFC approach allowed for a within-subject investigation of the degree to which temporal fluctuations in amygdala-mPFC FC tracks larger-scale fluctuations in the network. Here, the amygdala and mPFC ROIs fall within the limbic and DMNs, respectively, thus there are three within-subject time courses of interest: (1) amygdala-mPFC FC, (2) limbic – DMN FC, and (3) modularity *Q*-values. This is illustrated in **Figure 2** for a single representative participant. These time courses are then correlated and fisher *r*-to-*z* transformed within each subject, allowing group-level analyses of the distributions of the correlations and whether they differ as a function of early life trauma. We tested whether these relationships differed between the assaulted and control participants with multiple regression analyses, in which a dummy coded regressor representing assault exposure and the covariates of age, verbal IQ, and mean FD were entered as simultaneous predictors of the group-level distributions of Fisher *r*-to-*z*-transformed correlations.

#### ROI Specificity Analyses

To examine the specificity of any identified network relationships with the left amygdala, additional analyses were conducted using the bilateral caudate as an alternative ROI within the limbic network. In parallel to analyses using the left amygdala specifically, identical analyses were conducted with a left (ROI # 38 in the parcellation mask) and right (ROI # 198 in the parcellation mask) caudate ROI. The time courses of the left and right caudate were collapsed into a mean time course given that there was no *a priori* reason to select either the right or left caudate.

#### Exploratory Analysis of Network–Network Interactions Related to Amygdala-mPFC Connectivity

Finally, given that analyses here were mostly focused on understanding the relationships between amygdala-mPFC connectivity as a function of limbic-DMN connectivity, a complimentary analysis was conducted to test the degree to which amygdala-mPFC is attributable to other network–network interactions. In this approach, linear support vector regression (SVR) is used on a within-subject basis to quantify the degree to which all network–network interactions (10 × 10 network connectivity matrix = 45 unique network–network interactions) across the 41 time windows were predictive of the degree of amygdala-mPFC FC. Variables were *z*-scored prior to analyses, the SVR used a cost function = 1 implemented with LIBSVM (Chang and Lin, 2011), and performance was defined as the correlation between model predicted amygdala-mPFC FC vs observed amygdala-mPFC FC using a leave-one-out cross-validation approach. For each participant, model performance (correlation coefficient) and feature weights associated with each network–network interaction, which represent the degree to which the feature contributes to the SVR decision function, were stored for subsequent group-level analyses.

## Results

### Modular Brain Organization

The group-level community structure detected by the Louvain algorithm included 10 functional networks (**Figure 1**) corresponding to lateral visual, default mode, medial visual, limbic, posterior insula/somatosensory, bilateral superior temporal sulci, frontoparietal, motor, salience, and precuneus networks.

### Amygdala-mPFC FC and Large-Scale Network Modularity as a Function of Early Life Trauma

**FIGURE 3:**
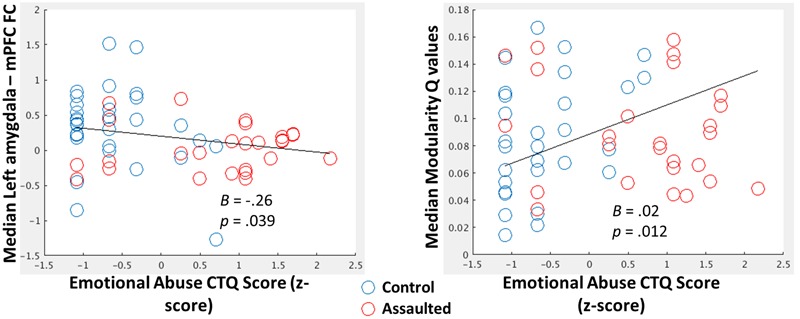
**(Left)** Scatterplot depicting the negative relationship between the emotional abuse CTQ subscale score (log-transformed and *z*-scored) and median within-subject FC between the amygdala and mPFC. **(Right)** Scatterplot depicting the positive relationship between the emotional abuse CTQ subscale score (log-transformed and z-scored) and median within-subject *Q*-values **(Right)**. For both scatterplots, B coefficients and *p*-values come from regression models in which all CTQ subscales, along with age, verbal IQ, and head motion, were entered as covariates.

The median FC between the amygdala and mPFC across windows was significantly negatively correlated with the emotional abuse subscales of the CTQ (**Figure 3**; Left) when controlling for age, verbal IQ, and head motion (mean FD across the scan).

The median modularity *Q*-values across windows was significantly positively correlated with the emotional abuse subscale of the CTQ (**Figure 3**; Right) when controlling for age, verbal IQ, and head motion.

### Role of Amygdala-mPFC FC within Larger Network Patterns

**FIGURE 4:**
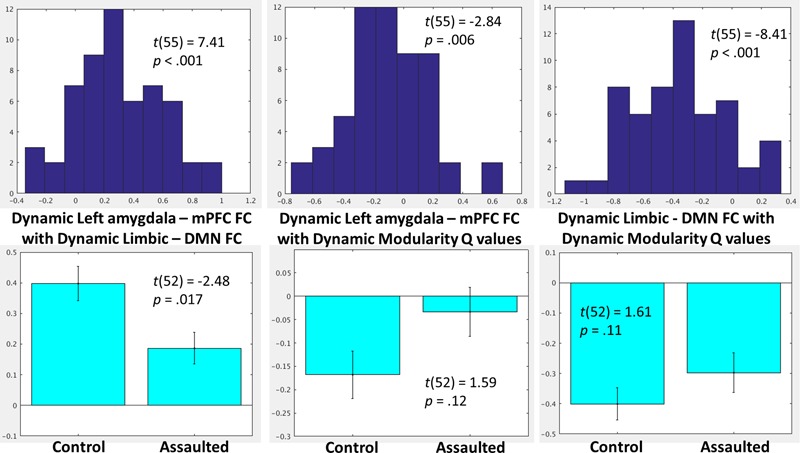
**(Top)** Histograms depicting the group-level distributions of the within-subject relationships between the amygdala-mPFC FC and limbic-DMN FC **(Top Left)**, amygdala-mPFC FC and modularity *Q*-values **(Top Middle)**, and limbic-DMN FC and modularity *Q*-values **(Top Right)**. **(Bottom)** Comparisons in the corresponding indices between the assaulted and control participants. The *x*-axis of the top histograms all correspond to fisher *r*-to-*z* transformed correlation coefficients between the network indices, which corresponds directly to the *y*-axis in the bottom bar graphs.

To investigate the role of the bi-nodal FC between the amygdala and mPFC within larger network patterns, the group-level distributions (**Figure 4**) were examined for the within-subject correlations between (1) dynamic amygdala-mPFC FC with dynamic limbic – DMN FC, (2) dynamic amygdala-mPFC FC with dynamic modularity *Q*-values, and (3) dynamic limbic – DMN FC with dynamic modularity *Q*-values. Group-level *t*-tests demonstrated that within-subject fluctuations in amygdala-mPFC tracked positively with the connectivity between the modules in which these ROIs belong, such that the amygdala and mPFC were more connected when the limbic and DMNs were more connected. By contrast, the amygdala and mPFC were less connected when overall network modularity was greater, and similarly the limbic and DMNs were less connected when the overall network modularity was greater.

### The Role of Amygdala-mPFC FC within Larger Network Patterns as a Function of Childhood Trauma

It was next tested whether the role of the bi-nodal amygdala-mPFC FC in the larger network patterns differed between assaulted and control adolescent girls. All subsequent analyses controlled for age, verbal IQ, and head motion. It was observed that assaulted adolescent girls demonstrated lessor correspondence between left amygdala-mPFC FC and dynamic limbic-DMN FC, though the direction of the effect was the same in both groups (**Figure 4**). There were not differences between groups in the correspondence between dynamic limbic-DMN FC and modularity *Q*-values or in the correspondence between amygdala-mPFC FC and modularity *Q*-values.

### Testing the Association between Caudate-mPFC FC and Network Organization

As a test of the specificity of observed relationships between amygdala-mPFC FC and network organization, parallel analyses were conducted with functional connectivity between the caudate and mPFC.

**FIGURE 5:**
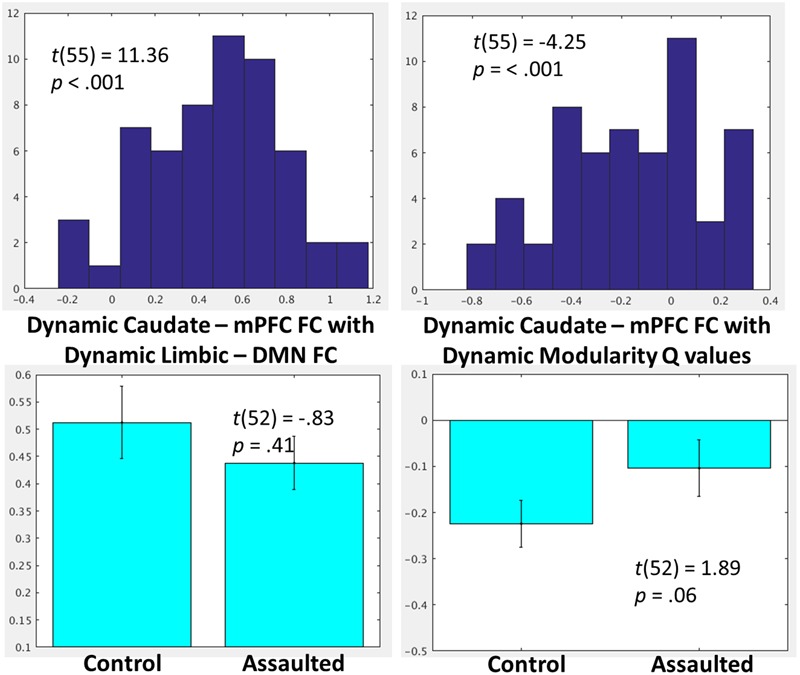
**(Top)** Histograms depicting the group-level distributions of the within-subject relationships between the caudate-mPFC FC and the limbic-DMN FC and the caudate-mPFC FC with modularity *Q*-values. **(Bottom)** Comparisons in the corresponding indices between the assaulted and control participants. The *x*-axis of the top histograms all correspond to fisher *r*-to-*z* transformed correlation coefficients between the network indices, which corresponds directly to the *y*-axis in the bottom bar graphs.

As indicated in **Figure 5**, and similar to the network patterns identified for FC between the amygdala and mPFC, the degree of FC between caudate and mPFC was also significantly related to degree of limbic-DMN FC as well as with degree of overall modularity *Q*-values.

The relationship between caudate-mPFC FC, dynamic limbic-DMN FC, and dynamic modularity *Q*-values were then compared between groups. There was no evidence of group differences in degree of correspondence between dynamic caudate-mPFC FC and dynamic limbic-DMN FC. There was only a trend for a lessor relationship between caudate-mPFC with modularity *Q*-values among assaulted compared to control girls (**Figure 5**).

### Exploratory Analysis of Dynamic Network–Network Interactions that Scale with Amygdala-mPFC Connectivity

**FIGURE 6:**
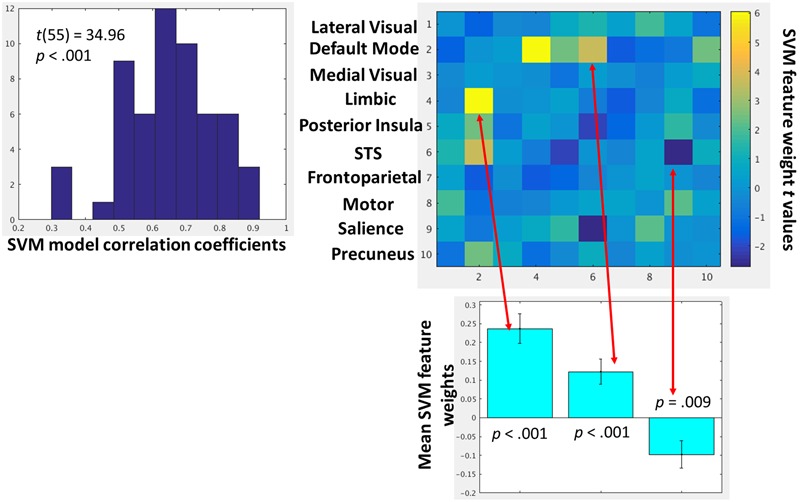
**(Left)** Histogram depicting the group-level distributions of the support vector regression model fits for each participant. The model fit indices are the pearson correlation coefficients between the observed amygdala-mPFC FC and model predicted values. **(Right)** Heat map depicting *t*-values of the one-sample *t*-tests on the support vector regression feature weights for each network–network interactions. These *t*-values represent how strongly, and in which direction, each network–network interaction predicted amygdala-mPFC FC across participants. Network–network interactions that were strongly predictive (*p* < 0.01) of amygdala-mPFC FC are further depicted in the below bar graphs.

The within-subject SVR analyses demonstrated excellent model fit, defined by leave-one-out cross-validation, across participants (**Figure 6**). One-sample *t*-tests were then conducted on the feature weights to identify which network–network interactions were consistently predictive of amygdala-mPFC FC across participants. A heat map of all one-sample *t*-test *t*-values is provided in **Figure 6**. Using a threshold of *p* < 0.01 given the exploratory nature of this network-wide analysis, there were only three network–network interactions consistently predictive of dynamic amygdala-mPFC FC: limbic-DMN FC, DMN-superior temporal sulcus FC, and salience-superior temporal sulcus FC. Whereas limbic-DMN FC and DMN-superior temporal sulcus FC had positive relationships with amygdala-mPFC FC, salience-superior temporal sulcus FC was negatively related to amygdala-mPFC FC. Notably, there were no significant interactions with the frontoparietal network that were related to amygdala-mPFC FC.

## Discussion

The purpose of this study was to investigate the degree to which a specific bi-nodal functional connectivity pattern, amygdala-mPFC, was associated with larger-scale network patterns. The commonly reported finding and predicted observation based on neurocircuitry models of trauma and PTSD of weakened static FC between amygdala-mPFC and childhood trauma (Rauch et al., 2006; Burghy et al., 2012; Herringa et al., 2013) was replicated in the current sample, such that the median FC between the amygdala and mPFC across the time windows was negatively correlated with the continuous emotional abuse subscale of the CTQ, as has been previously reported (Dannlowski et al., 2012). Additionally, it was also observed that greater emotional abuse was associated with heightened modularity *Q*-values. Given that modularity *Q*-values are defined based on the patterns of within- vs. between-network connections, and that the amygdala and mPFC belong to different networks, it would be expected that early life trauma would have opposing relationships with these two indices of brain function. Indeed, these opposing relationships beg the question of how, on a within-subject basis, bi-nodal FC relates to larger-scale patterns of brain function.

With respect to the role of the bi-nodal amygdala-mPFC FC within the larger-network patterns, it was observed that this bi-nodal FC closely tracked both the FC of the networks to which the nodes belong as well as to the larger-network patterns. Moreover, the observed effects were in the expected directions from a network-level perspective. That is, the amygdala and mPFC ROIs belong to the limbic and DMN sub-networks, respectively, and as such, it would be expected that the bi-nodal FC track the overall degree of FC between these networks. Similarly, network modularity *Q*-values are defined by the degree to which edges lie within, as opposed to between, communities, and as such, it would be expected that as modularity *Q*-values increase, FC between nodes in different modules would necessarily decrease. These group-level findings ostensibly confirm the larger concept that perhaps one should not interpret a single bi-nodal FC pattern without considering the modules in which the nodes belong and the larger network patterns. From this perspective, one might re-conceptualize weakened amygdala-mPFC FC, such that instead of inferring that weakened FC between these nodes suggests a weakened inhibitory effect of the mPFC on the amygdala, one might instead infer less overall communication between the DMN and the limbic system or greater overall network partitioning. This difference in inference would seemingly have a significant downstream impact on clinical efforts to correct/modify the observed bi-nodal FC findings, such as targeting the larger network patterns rather than targeting anything specific about amygdala-mPFC connectivity.

Nonetheless, it is relevant to note the results of the control analyses with the caudate. On the one hand, the network analyses demonstrated similar relationships between caudate-mPFC FC with the limbic-DMN FC and overall modularity *Q*-values, supporting the inference that bi-nodal FC is related to larger-scale network patterns. On the other hand, there was no evidence that the caudate-mPFC FC differed between groups. The latter result suggests specificity of the effect of early life trauma on amygdala-mPFC FC and that there is not a generic relationship between any node in the limbic network with the DMN. Future research is clearly needed to continue to probe the degree of bi-nodal FC independence from larger network function, and the associated impact of early life trauma.

As an exploratory analysis, a complimentary data-driven approach was used to characterize which network–network interactions were consistently predictive of degree of amygdala-mPFC connectivity. This approach demonstrated three network–network interactions consistently related to amygdala-mPFC FC: (1) as expected, the limbic-DMN FC was positively predictive, (2) the DMN-superior temporal sulcus FC was positive predictive, while (3) the salience-superior temporal sulcus FC was negatively predictive. There was no evidence that interactions with the frontoparietal network were consistently related to degree of amygdala-mPFC FC. Further, the limbic-DMN FC clearly, and intuitively, was the strongest predictor and the only relationship that would survive correction for multiple comparisons if set stringently with FDR. These network-wide exploratory analyses seemingly confirm inferences from the *a priori* analyses and demonstrate the importance of understanding bi-nodal FC within the context of the networks in which the individual nodes belong.

There was not consistently strong evidence that the magnitude of cross-level analyses differed between the assaulted and control participants. There was a significantly lesser relationship between amygdala-mPFC FC and limbic-DMN FC among the assaulted girls, and there appeared to be trends toward similar effects on the other cross-level analyses. Nonetheless, the direction of the relationships (e.g., positive relationships between amygdala-mPFC FC and limbic-DMN FC) was consistent in each group, suggesting that the qualitative nature of the relationships did not differ between groups. These within-group effects potentially reinforce the inferences made in the preceding paragraph regarding the importance of interpreting bi-nodal FC within the context of larger-scale network organization.

While the data presented here shed light on the role of a canonical bi-nodal FC findings within larger network patterns and also on how early life trauma might disrupt or alter the role a bi-nodal FC pattern within the larger network, the current study is not without limitations. First, the sample is limited to adolescent girls, and the degree to which the findings generalize to male samples or adult samples needs to be addressed. Second, the cross-sectional nature of the design precludes any causal inferences regarding the relationship between early life trauma exposure and alterations in bi-nodal FC or larger network patterns. Third, a relatively small parcellation of 250 ROIs was used, whereas prior research from this lab has used a larger 824 ROI parcellation (Cisler et al., 2016). The reason for this is the use of the DFC approach, such that repeating the community detection algorithm on an 824 × 824 matrix with 25 iterations at each of the 41 time windows for each of the 56 participants was computationally expensive. In support of the smaller parcellation approach used here, we observed networks that were highly similar, though not identical, to that observed in the previous report. Nonetheless, it remains possible that different results regarding the role of amygdala-mPFC FC within larger network patterns might be different if the larger network patterns were defined using a larger parcellation. Future research is clearly still needed to continue to understand how to interpret bi-nodal FC patterns within the context of a network-level conceptualization of human brain function.

## Ethics Statement

The studies from which these data were collected were approved by the University of Arkansas for Medical Sciences IRB. A parent or caregiver provided written consent and all adolescents provided written assent.

## Author Contributions

JC was involved in the design, analysis, interpretation, and writing of this manuscript.

## Conflict of Interest Statement

The author declares that the research was conducted in the absence of any commercial or financial relationships that could be construed as a potential conflict of interest.

## Supplementary Material

FIGURE S1 **Plot of the average Rand *Z*-scored index of similarity across each partition (*y*-axis) as a function of the gamma resolution parameter (*x*-axis) used by the Louvain community detection algorithm**.
