# Supplementary figures and images for: Childhood Trauma and Functional Connectivity between Amygdala and Medial Prefrontal Cortex: A Dynamic Functional Connectivity and Large-Scale Network Perspective
Source: Front Syst Neurosci. 2017 May 11;11:29. doi: 10.3389/fnsys.2017.00029 (PMC5425605; doi:10.3389/fnsys.2017.00029)

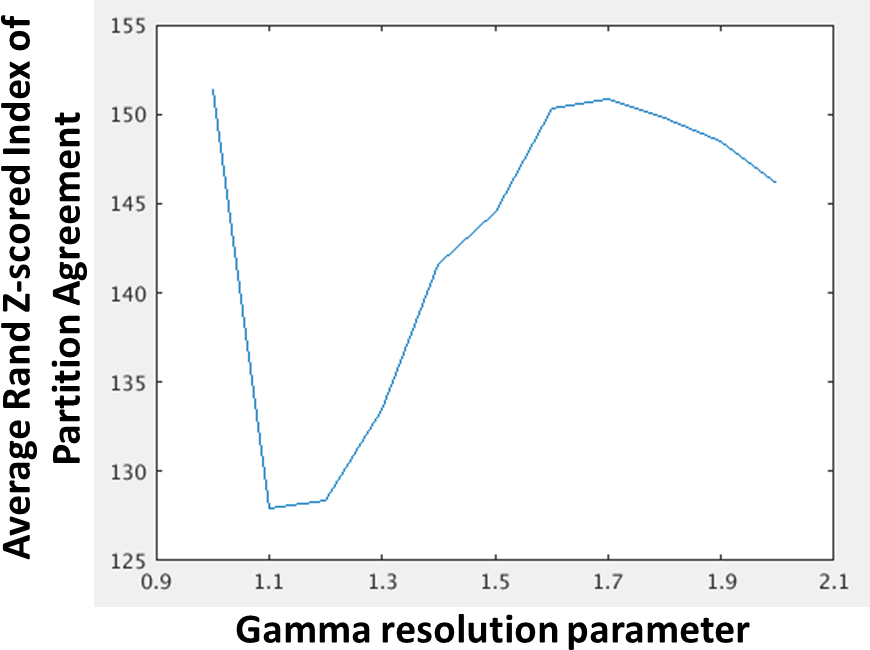

Supplement: FIGURE S1 — Plot of the average Rand Z-scored index of similarity across each partition (y-axis) as a function of the gamma resolution parameter (x-axis) used by the Louvain community detection algorithm. [file Image_1.TIF]
